# Portuguese Migrants in Switzerland: Healthcare and Health Status Compared to Portuguese Residents

**DOI:** 10.1371/journal.pone.0077066

**Published:** 2013-10-08

**Authors:** Luís Alves, Ana Azevedo, Henrique Barros, Fred Paccaud, Pedro Marques-Vidal

**Affiliations:** 1 Instituto de Saúde Pública da Universidade do Porto, Porto, Portugal; 2 Departamento de Epidemiologia Clínica, Medicina Preditiva e Saúde Pública, Porto, Portugal; 3 Unidade de Saúde Familiar St. André de Canidelo, Vila Nova de Gaia, Portugal, Faculdade de Medicina da Universidade do Porto, Porto, Portugal; 4 Institute of Social and Preventive Medicine (IUMSP), Lausanne University Hospital, Lausanne, Switzerland; Iran University of Medical Sciences, Islamic Republic of Iran

## Abstract

**Background:**

Most migrant studies have compared health characteristics between migrants and nationals of the host country. We aimed at comparing health characteristics of migrants with nationals from their home country.

**Methods:**

Portuguese national health survey (2005-6; 30,173 participants aged 18-75 years) and four national health surveys conducted in Switzerland (2002, 2004, 2007 and 2011, totalling 1,170 Portuguese migrants of the same age range). Self-reported data on length of stay, cardiovascular risk factors, healthcare use and health status were collected.

**Results:**

Resident Portuguese were significantly older and more educated than migrants. Resident Portuguese had a higher mean BMI and prevalence of obesity than migrants. Resident Portuguese also reported more frequently being hypertensive and having their blood pressure screened within the last year. On the contrary, migrant Portuguese were more frequently smokers, had a medical visit in the previous year more frequently and self-rated their health higher than resident Portuguese. After adjustment for age, gender, marital status and education, migrants had a higher likelihood of smoking, of having a medical visit the previous year, and of self-rating their current health as good or very good than resident Portuguese. Compared to Portuguese residents, cholesterol screening in the previous year was more common only among migrants living in Switzerland for more than 17 years.

**Conclusion:**

Portuguese migrants in Switzerland do not differ substantially from resident Portuguese regarding most cardiovascular risk factors. Migrants consider themselves healthier than Portuguese residents and more often had a recent medical visit.

## Introduction

In 2010, one in three of all international migrants in the world lived in Europe, representing 8.7% of the total European population [1]. Migrants tend to exhibit disadvantaged cardiovascular risk factor profiles, namely obesity and hypertension [2]. Thus, migrants usually constitute a vulnerable group and may benefit from specific preventive strategies which tend to differ from those relevant to the host population.

Most migrant studies compared migrants and nationals from the host country [3–7], but the comparison of migrant populations with the population of their country of origin is of importance because it permits assessing the effect of the change in living and working conditions on diverse health parameters. Still, most studies focused on migrants going from a developing country to a developed one, and less work has been done regarding migrants going from a developed country to another [8].

Switzerland is multicultural country with approximately 8 million inhabitants that has one of the highest rates of foreigners in Europe. In 2011, approximately 215,000 Portuguese had a permanent residency in Switzerland, representing 12.3% of the total foreign population [9]. Although some studies have compared the health status of Portuguese migrants with Swiss nationals [5,6,10], little is known whether cardiovascular risk profiles and related healthcare utilization differ between Portuguese migrants in Switzerland and resident Portuguese. Hence, this study aimed to compare cardiovascular risk factor prevalence, healthcare use and self-perceived health between resident Portuguese and Portuguese migrants in Switzerland.

## Methods

### Data sources

Data from the Portuguese National Health Survey (2005/6) was provided by Statistics Portugal upon request. The National Health Survey is a cross-sectional, nationwide population-based survey and its methodology has been described previously [11,12]. The survey was conducted between February 2005 and January 2006. The primary sampling unit was the house, and data were derived from the population and housing national census. Within each main geographical region, two strata were defined: the parishes and, within parishes, geographically defined units of approximately 240 residences. The houses were then randomly selected within each geographically defined unit. All subjects living in the house were surveyed. The survey was carried out in compliance with the Helsinki Declaration. Trained interviewers collected data according to a standardized protocol [11] in personal computer-assisted face-to-face interviews. Quality control was performed by reapplying the same questionnaire to 10% of the initial sample. Different interviewers reapplied the questionnaire and the time between interviews did not exceed 3 weeks. Participation rate (percentage of households who responded) was 76%, as reported by Statistics Portugal. Migrants, subjects aged less than 18 or over 75 years or with missing data for gender, marital status or education were excluded from this analysis. Of the initial 41,193 participants, 30,173 (73.2%) were retained for analysis.

Data from the Swiss Health Surveys of 2002 and 2007 were obtained from the Swiss Federal Statistical Office. The Swiss Health Survey is a cross–sectional, nationwide, population–based telephone survey conducted every 5 years since 1992 by the Swiss Federal Statistical Office under a mandate from the federal government [13]. The surveys can be considered as representative of the Swiss population. The study population was selected by stratified random sampling of all private Swiss households with fixed-line telephones. The first sampling stratum consisted of the seven main geographic regions. The second stratum consisted of the cantons. The number of sampled households was proportional to the population size of the canton. In order to obtain accurate cantonal estimates, oversampling of households was performed in some cantons. The third stratum consisted of the households. Within each household, one member aged 15 years or more was randomly selected. A letter inviting for participation in the survey was sent to each sampled subject and computer–assisted telephone interviews were performed using software to manage dialling and data collection. The interviews were carried out in German, French or Italian as appropriate. People who did not speak any of these three languages were excluded from the survey. Subjects seeking asylum or living in a nursing home were also excluded [13]. Elderly subjects, aged above 75 years, were offered the possibility of answering in a face-to-face interview instead of telephone, and in the case of people who were unable to reply themselves, due to illness, handicap, long absence or language problems, a close person was interviewed instead. Participation rates were 64% in 2002 and 66% in 2007. Participants aged less than 18 or over 75 years, or with missing data for gender, marital status or education were excluded. In the 2002 survey 154 Portuguese migrants were interviewed and 209 in 2007, of which 114 (74.0%) and 208 (99.5%) had complete data.

Data from two nationwide migrant studies commissioned by the Swiss Federal Office of Public Health were also used. The first migrant study (GMM1) was conducted in 2004, the second (GMM2) in 2010. The sample was obtained from population registers in GMM1 and from the central register of immigration in 2010. In GMM1, sample stratification was performed by gender and age group and migrants living in Switzerland for less than one year were excluded [14]. In GMM2, sample stratification was performed by gender, age group, country of birth and length of stay in Switzerland; within each stratum, inhabitants were randomly selected to obtain a minimum of 50 individuals [15]. For both surveys, data was collected by telephone interview in French, German or in the migrant’s language (including Portuguese). The structured questionnaires were translated to Portuguese from the original German version and back-translated to German to assess the quality of the translations. Participation rates were 68.4% in GMM1 and 37.9% in GMM2. Participants aged less than 18 or over 75 years, or with missing data for gender, marital status or education were excluded. In GMM1 511 Portuguese migrants were interviewed and 450 in GMM2, of which 445 (87.1%) and 403 (89.5%) had complete data for analysis.

### Collected data

From all surveys, self-reported data were collected on gender, age, marital status, education, weight, height, smoking status, hypertension status, last blood pressure (BP) screening, last cholesterol screening, last medical visit and general self-perceived health.

Age was categorized as 20-39, 40-59 and 60-79 years. Marital status was coded as living in couple (married/living together) or single (single, divorced, separated or widowed). Education was classified as elementary, secondary or higher education. Body mass index (BMI) was calculated from weight (in kilograms) and height (in meters). Hypertension was considered whenever the participant answered “Yes” the specific question or when reported having had a treatment with anti-hypertensive drugs over the last two weeks. Information on this variable was not available for GMM1 and GMM2. Information of last BP screening was dichotomized as having had at least one BP screening in the last year or not. This variable was collected only during the weeks 27 to 39 in the Portuguese health survey and was not available for GMM2. Information of last cholesterol screening was dichotomized as having at least one cholesterol screening in the last year or not. This variable was collected only during the Portuguese health survey weeks 27 to 39 and was not available for GMM2. Data on the last medical visit was categorized as having had at least a medical visit in the previous year or not. Self-reported health was coded as “very good” or “good” vs. “fair”, bad” and “very bad”. Finally, length of stay was available for 550 participants in the Swiss Health Survey of 2002 and in the GMM1.

### Statistical analysis

Statistical analysis was conducted using Stata v.12 (Stata Corp, College Station, TX, USA). Data from Portuguese migrants in Switzerland was pooled from the two Swiss Health Surveys and from the two Swiss Migrant Surveys. Continuous and categorical variables were expressed as mean ± standard deviation or number of participants (percentage), respectively. Length of stay in Switzerland was also expressed as median (interquartile range). Bivariate comparisons were performed using Student t-test for continuous variables or Chi-square test for categorical variables. Multivariate analysis was performed using logistic regression, adjusting for gender, age group, marital status and education. Tests for interaction between education and migration status were performed. Statistical significance was considered for p<0.05.

## Results

### Sample description

The main characteristics of the Portuguese migrants and of their resident counterparts are summarized in table 1. Migrants were significantly younger and less educated than resident Portuguese, while no differences were observed for gender or marital status. Similar findings were obtained when the analysis was stratified on gender (not shown).

**Table 1:** Characteristics of the samples according to immigration status.

		**Resident**	**Migrants**	**p-value**
		**(n=30,173)**	**(n=1,170)**	
**Gender**	Woman	15,770 (52.3)	587 (50.2)	
	Man	14,403 (47.7)	583 (49.8)	0.16
**Age (years)**	20-39	9,521 (31.5)	562 (48.0)	
	40-59	11,307 (37.5)	489 (41.8)	<0.001
	60-79	9,345 (31.0)	119 (10.2)	
**Marital status**	Living in couple	21,269 (70.5)	854 (73.0)	
	Single §	8,904 (29.5)	316 (27.0)	0.07
**Employment status**	Employed	17,217 (57.1)	878 (75.0)	
	Other	12.956 (42.9)	292 (25.0)	<0.001
**Education**	Elementary	23,592 (78.2)	824 (70.4)	
	Secondary	3,388 (11.2)	284 (24.3)	<0.001
	Higher	3,193 (10.6)	62 (5.3)	

Results are expressed as number of participants (percentage). § single, divorced or widowed. Statistical analysis by chi-square test.

Among migrants, mean length of stay was 19±9 years, with a median of 17 years and an interquartile range of 14–23 years.

### Cardiovascular risk factors prevalence and management

The bivariate comparisons of cardiovascular risk factor prevalence and screening between Portuguese migrants and residents are summarized in table 2. Overall, resident Portuguese had a higher mean BMI and prevalence of obesity than migrants. Resident Portuguese also reported more frequently being hypertensive and having their blood pressure screened within the last 12 months. On the contrary, migrant Portuguese were more frequently smokers, had a medical visit in the previous year more frequently and self-rated their health as good or very good more frequently than resident Portuguese. No differences were found between resident and migrant Portuguese regarding cholesterol screening.

**Table 2:** Cardiovascular risk factors prevalence and screening, and health care utilization, by migrant status.

		**Resident**	**Migrants**	**p-value**
		**n=29,637**	**n=1,151**	
BMI (Kg/m^2^)		26.0 ± 4.4	25.3 ± 4.0	<0.001
BMI groups (%)	Normal	13,413 (45.3)	601 (52.2)	
	Overweight	11,312 (38.2)	401 (34.8)	<0.001
	Obese	4,912 (16.7)	149 (13.0)	
		n=30,161	n=1,170	
Current smoking (%)		6,520 (21.6)	344 (29.4)	<0.001
		n=30,156	n=310	
Hypertension (%)		7,919 (26.3)	48 (15.5)	<0.001
		n=7,427	n=557	
BP screening (%)§		6,026 (81.1)	425 (76.3)	<0.01
		n=7,391	n=555	
Cholesterol screening (%)§		4,812 (65.1)	351 (63.2)	0.38
		n=21,834	n=1,169	
Medical visit (%)§		7,792 (35.7)	903 (77.2)	<0.001
		n=21,075	n=1,169	
Health status good/very good (%)		8,278 (39.3)	849 (72.6)	<0.001

Results are expressed as average ± standard deviation or as number of participants (percentage). § during the previous 12 months. Statistical analysis by Student’s t-test or chi-square.

The results from the multivariate analyses are presented in table 3. All estimates were adjusted for gender, age group, marital status and education. Migrants had a higher likelihood of being smokers, of having a medical visit the previous 12 months, and of self-rating their current health as good or very good than resident Portuguese. No differences were found between resident and migrant Portuguese for obesity, hypertension, BP and cholesterol screening.

**Table 3:** Multivariate analysis of the association between migration status and length of stay and cardiovascular risk factors or health care use.

	**Migrant status**			**Migrant status, according to length of stay §§**				
	**Portugal**	**Switzerland**		**Portugal**	**Switzerland, ≤17 years**		**Switzerland, >17 years**	
Obesity (n=30,778)	1 (ref.)	0.87	(0.73-1.04)	1 (ref.)	0.77	(0.52-1.13)	0.85	(0.61-1.19)
Current smoking (n=31,331)	1 (ref.)	1.18	(1.03-1.36)	1 (ref.)	1.22	(0.93-1.60)	0.98	(0.72-1.34)
Hypertension (n=30,466)	1 (ref.)	1.11	(0.80-1.54)	1 (ref.)	1.73	(0.88-3.41)	0.60	(0.22-1.61)
Blood pressure screening§ (n=7984)	1 (ref.)	0.9	(0.72-1.11)	1 (ref.)	1.05	(0.78-1.41)	0.86	(0.62-1.21)
Cholesterol screening § (n=7946)	1 (ref.)	1.07	(0.89-1.30)	1 (ref.)	1.03	(0.79-1.35)	1.36	(1.02-1.82)
Medical visit § (n=23,003)	1 (ref.)	9.2	(7.96-10.6)	1 (ref.)	10.1	(7.54-13.5)	7.23	(5.35-9.77)
Health status good/very good (n=22,244)	1 (ref.)	2.76	(2.39-3.18)	1 (ref.)	2.08	(1.57-2.75)	3.44	(2.62-4.50)

Results are expressed as Odds ratios and 95% confidence interval obtained with logistic regression models adjusted for age, marital status and education. § in the previous 12 months; §§ Analysis restricted to Portuguese residents and 550 Portuguese migrants with information on length of stay.

### Effect of length of stay

The effect of length of stay in Switzerland in prevalence and management of cardiovascular risk factors is summarized in table 3. After multivariate adjustment, no difference was found regarding the association with the prevalence of obesity and hypertension, and hypertension screening. When compared to Portuguese residents, only migrants living for more than 17 years in Switzerland had a higher likelihood of having their blood cholesterol screened in the previous year. All migrants (irrespective of their length of stay) had a higher likelihood of having a medical visit the last 12 months, although the magnitude of this association decreased with the length of stay. Finally, all migrants had a higher likelihood of reporting a “very good” or “good” health status and the magnitude of this association increased with the length of stay.

## Discussion

This is one of the very few studies that focused on migrants coming from one developed country to another. This is also the first study comparing supposedly representative samples of resident Portuguese and Portuguese migrants in Switzerland.

### Sample characteristics

Migrants were younger and less educated than resident Portuguese. These findings can be explained by the fact that most migrants come to Switzerland for work, and older people will face more difficulties in finding work. There might be also more opportunities for blue-collar work in Switzerland than in Portugal. This is supported by the fact that, in Portugal, subjects with lower educational level have higher unemployment rates: 11.7% for subjects with elementary education vs. 7.1% for subjects with university level (2010 data) [16].

### Cardiovascular risk factors prevalence and management

Migrants tended to be slimmer than resident Portuguese, but this difference disappeared after multivariate adjustment. These findings do not confirm a previous study comparing resident Greeks and migrant Greeks in Australia, where migrants presented a higher prevalence of obesity [17]. In another study, adult weight among Samoans who migrated to California greatly exceeded the weight of their counterparts in Hawaii and Samoa [18]. Still, Portuguese migrants present higher obesity levels than Swiss nationals [5]. Overall, our results suggest that Portuguese migrants do not differ from their resident counterparts regarding BMI and obesity levels, although other possible explanations such as differences in settings of migration cannot be ruled out.

Prevalence of smoking was higher in migrants, and this difference persisted after multivariate adjustment. Most likely this difference results from residual confounding by socioeconomic status, since education alone may not account for the whole socioeconomic effect on smoking. Additionally, the lack of national legislation regarding smoking bans in Switzerland may have contributed. Indeed, contrary to Portugal, smoking legislation is decided at the cantonal level in Switzerland, leading to considerable differences between cantons [19]. It is thus possible that Portuguese migrants might be less constrained regarding smoking in Switzerland.

The prevalence of self-reported hypertension was considerably lower among migrants, but this difference was no longer significant after multivariate adjustment. Migrants also reported a lower BP screening, but again this difference became nonsignificant after multivariate adjustment. These findings do not replicate the results from previous studies [18,20,21]. A possible explanation for this discrepancy is the fact that, contrary to developing countries, BP and hypertension levels are relatively high in Portugal, and might thus not change upon migration [22]. Still, in a study that assessed the prevalence and management of risk factors in migrant groups in Switzerland, Portuguese migrants reported less frequently being told they were hypertensive than Swiss nationals and also reported less frequently having their blood pressure screened [6].

No difference was found between migrant and resident Portuguese regarding cholesterol screening, and this was confirmed after multivariate adjustment. To the best of our knowledge, this is the first study that assessed cholesterol screening among migrants and resident subjects. Overall, our results suggest that Portuguese migrants in Switzerland benefit from the same level of cardiovascular risk factor screening than in their resident country.

Previous work on migrants’ use of healthcare in Europe showed that, compared with non-migrants, migrants tend to have more contacts with general practitioners [23]. In the present study, Portuguese migrants reported a higher frequency of medical visits in the previous year and this difference increased after multivariate adjustment. Still, this finding was somewhat unexpected and might be related to difference in health systems between Switzerland and Portugal. In Switzerland, the choice of the general practitioner is free, and a patient can have several family doctors. In Portugal, patients should register at a health centre, where they are attributed to a general practitioner; hence, obtaining an appointment might be more difficult in Portugal than in Switzerland, which might reduce the number of visits. However, further studies are needed to better assess this point, namely to take into account the appropriateness of use of care.

Interestingly, Portuguese migrants reported more frequently having a “very good” or “good” health status than resident Portuguese, and this difference was even increased after multivariate adjustment. The most likely explanation might be employment, a potent determinant of health status [24]. For instance, in 2010, the unemployment rates in Portugal and Switzerland were 11% and 3.7%, respectively. Although the overall Swiss unemployment rates are higher among foreigners, the Portuguese, the Spanish and the Germans have a lower risk of unemployment than the Italians or those from new immigration countries [25]. Other possible explanations include the incomplete adjustment for other potential confounders or a “healthy migrant effect”. However, the fact that this association was stronger among Portuguese migrants with a longer stay in Switzerland argues against the latter hypothesis. Still, we repeated our analyses after adjustment for employment status (see table S1) but did not observe significant differences from the initial analysis.

### Effect of length of stay

After multivariate adjustment, length of stay had no effect on the association between migrant status and the prevalence of obesity and hypertension.

Migrants who arrived in Switzerland more recently had a higher likelihood of having had a medical visit in the previous year. It is likely that young migrants who arrive in Switzerland benefit from the Swiss health system and mandatory occupational medicine examinations which would make them attend a medical visit more often. The slight time attenuation, though with older migrants still having a higher prevalence of a medical visit in the previous years than Portuguese residents, may be framed within the net result of both ageing (increasing use and needs) and growing confidence in the healthcare system, working in opposite directions.

Regarding the cardiovascular risk factor assessment, on one hand there were no differences in blood pressure screening, regardless of the length of stay; on the other hand, only migrants with a longer stay in Switzerland had a significantly higher likelihood of having had a blood cholesterol check in the previous year. The apparent contradiction may be related with the high prevalence of hypertension in the Portuguese population, with possibly many blood pressure measurements being related with previous awareness of high blood pressure and not true screening.

Finally, migrants with a longer length of stay rated their health status “good” or “very good” more frequently, and this association increased with the number of years in Switzerland: OR for one year increase 1.04 (1.01-1.07), p<0.005. This effect may reflect not only the objective health status but also an attitude towards health and the relation with expectations, both of which can change the subjective self-assessment of health status. However, we cannot exclude the possibility of migrants with health problems being more likely to return to their home country earlier, resulting in a partly artificial association.

### Strengths and limitations

The strengths of this study are that it was based on national surveys and thus the data can be considered as roughly representative of the populations considered. The sample sizes are relatively large compared to other studies using the same epidemiologic design [20,21], thus allowing a better estimation of the effects. We also used Swiss data from several periods encompassing the Portuguese Health Survey to take into account possible secular modifications in cardiovascular risk factor prevalence and/or management.

This study has also several limitations. First, selection bias of Portuguese migrants is a potential problem in the Swiss Health Surveys, as subjects who did not speak French, German or Italian were excluded. This could imply a bias in the direction of participants with higher education or who have been living in Switzerland long enough to learn the language. However, since the Swiss Migrant Surveys have been conducted in the participant’s mother tongue, we believe that this bias is reduced.

Second, only self-reported data were used, which might lead to an underestimation of obesity [26] and cardiovascular risk factor levels [22] but, as all studies relied on the self-reported data, it can be expected that the magnitude of the underestimation is similar and that no major bias is present. It has also been suggested that reliability of self-rated health is low, especially among disadvantaged sociodemographic groups [27]. However, self-rated health has been consistently associated with health behaviours and outcomes in multiple studies [28,29]. Furthermore, we systematically adjusted for education in our multivariate analyses.

Third, the participation rate of Portuguese migrants in GMM2 was only 37.9%, which may limit representativeness. Still, this value is close to the participation rates of other surveys conducted in Switzerland [30]. Further, among the 403 Portuguese migrants evaluated, 89.5% had complete data for analysis. Also, this specific survey contributed with only one third of observations of the overall sample of Portuguese migrants. As such, any possible systematic errors were diluted in the analyses.

## Conclusion

Portuguese migrants in Switzerland do not differ substantially from resident Portuguese regarding most cardiovascular risk factors and cardiovascular risk screening. Migrants consider themselves healthier than Portuguese residents and more often had a recent medical visit.

## Supporting Information

Table S1 **Multivariate analysis of the association between migration status and length of stay and cardiovascular risk factors or health care use.**
(DOCX)
