# Supplementary material for: Portuguese Migrants in Switzerland: Healthcare and Health Status Compared to Portuguese Residents
Source: PLoS One. 2013 Oct 8;8(10):e77066. doi: 10.1371/journal.pone.0077066 (PMC3792909; doi:10.1371/journal.pone.0077066)
Supplement: Table S1 — Multivariate analysis of the association between migration status and length of stay and cardiovascular risk factors or health care use. (DOCX) [file pone.0077066.s001.docx]

**Table S1**: Multivariate analysis of the association between migration status and length of stay and cardiovascular risk factors or health care use.

|  | **Migrant status** | | **Migrant status, according to length of stay §§** | | |
| --- | --- | --- | --- | --- | --- |
|  | **Portugal** | **Switzerland** | **Portugal** | **Switzerland, ≤17 years** | **Switzerland, >17 years** |
| Obesity (n=30,778) | 1 (ref.) | 0.88 (0.74-1.05) | 1 (ref.) | 0.78 (0.53-1.15) | 0.85 (0.61-1.18) |
| Current smoking (n=31,331) | 1 (ref.) | 1.17 (1.02-1.34) | 1 (ref.) | 1.21 (0.92-1.59) | 0.99 (0.72-1.35) |
| Hypertension (n=30,466) | 1 (ref.) | 1.14 (0.82-1.58) | 1 (ref.) | 1.79 (0.91-3.53) | 0.61 (0.22-1.64) |
| Blood pressure screening § (n=7984) | 1 (ref.) | 0.90 (0.72-1.12) | 1 (ref.) | 1.05 (0.78-1.42) | 0.86 (0.62-1.21) |
| Cholesterol screening § (n=7946) | 1 (ref.) | 1.08 (0.89-1.30) | 1 (ref.) | 1.04 (0.79-1.36) | 1.36 (1.02-1.82) |
| Medical visit § (n=23,003) | 1 (ref.) | 9.46 (8.17-11.0) | 1 (ref.) | 10.1 (7.54-13.5) | 7.23 (5.35-9.77) |
| Health status good/very good (n=22,244) | 1 (ref.) | 2.70 (2.34-3.12) | 1 (ref.) | 2.02 (1.53-2.68) | 3.50 (2.67-4.59) |

Results are expressed as Odds ratios and 95% confidence interval obtained with logistic regression models adjusted for age, marital status, education and employment status. §, in the previous 12 months; §§, Analysis restricted to Portuguese residents and 550 Portuguese migrants with information on length of stay.
